# REMARRY and PURSUIT trials: liquid biopsy-guided rechallenge with anti-epidermal growth factor receptor (EGFR) therapy with panitumumab plus irinotecan for patients with plasma *RAS* wild-type metastatic colorectal cancer

**DOI:** 10.1186/s12885-021-08395-2

**Published:** 2021-06-07

**Authors:** Hiromichi Nakajima, Daisuke Kotani, Hideaki Bando, Takeshi Kato, Eiji Oki, Eiji Shinozaki, Yu Sunakawa, Kentaro Yamazaki, Satoshi Yuki, Yoshiaki Nakamura, Takeharu Yamanaka, Takayuki Yoshino, Takashi Ohta, Hiroya Taniguchi, Yoshinori Kagawa

**Affiliations:** 1 Department of Gastrointestinal Oncology, National Cancer Center Hospital East, Kashiwa, Japan; 2 Department of Clinical Oncology, Aichi Cancer Center Hospital, Nagoya, Japan; 3 Department of Surgery, National Hospital Organization Osaka National Hospital, Osaka, Japan; 4 Department of Surgery and Science, Graduate School of Medical Sciences, Kyushu University, Fukuoka, Japan; 5 Department of Gastroenterology, The Cancer Institute Hospital of the Japanese Foundation for Cancer Research, Tokyo, Japan; 6 Department of Clinical Oncology, St. Marianna University School of Medicine, Kawasaki, Japan; 7 Divison of Gastrointestinal Oncology, Shizuoka Cancer Center, Shizuoka, Japan; 8 Department of Gastroenterology and Hepatology, Hokkaido University Hospital, Sapporo, Japan; 9 Department of Biostatistics, Yokohama City University School of Medicine, Yokohama, Japan; 10 Department of Clinical Oncology, Kansai Rosai Hospital, Amagasaki, Hyogo Japan; 11 Department of Colorectal Surgery, Osaka General Medical Center, 3-1-56 Bandai-Higashi, Sumiyoshi-ku, Osaka, Japan

**Keywords:** Metastatic colorectal cancer, Circulating tumor DNA, Liquid biopsy, Rechallenge, Anti-EGFR mAb

## Abstract

**Background:**

Previous clinical trials have demonstrated the potential efficacy of rechallenge with anti- epidermal growth factor receptor (EGFR) monoclonal antibodies (mAbs) for patients with *RAS*/*BRAF* V600E wild-type metastatic colorectal cancer (mCRC). Moreover, post hoc biomarker analyses of clinical trials has suggested that *RAS* status in circulating tumor DNA (ctDNA) has a high probability to select patients who could benefit from anti-EGFR mAb rechallenge.

**Methods:**

This trial is composed of 2 phases: a monitoring phase (REMARRY) and a trial phase (PURSUIT). A monitoring phase, the REMARRY study, aims to evaluate the dynamics of plasma *RAS* status during the subsequent treatments after refractory to anti-EGFR therapy in patients with mCRC with *RAS*/*BRAF* V600E wild-type tumors who have progressed after a response to previous anti-EGFR therapy, using a highly sensitive digital polymerase chain reaction OncoBEAM RAS CRC kit in a central laboratory (Sysmex, Japan). A trial phase, the PURSUIT trial, is a multicenter, single-arm phase II trial to assess the efficacy and safety of rechallenge therapy with panitumumab plus irinotecan in patients without *RAS* mutations in ctDNA (plasma *RAS* negative) in the REMARRY study. Key eligibility criteria of the PURSUIT trial include *RAS*/*BRAF* V600E wild-type mCRC in tumor tissue refractory or intolerant to fluoropyrimidine, oxaliplatin, and irinotecan; progression after complete or partial response to previous anti-EGFR therapy; plasma *RAS* negative (defined as plasma mutant allele frequencies [MAF] of all *RAS* ≤ 0.1%) within 28 days prior to enrollment; 4 months or more between the last administration of previous anti-EGFR mAb and the start of protocol treatment; and Eastern Cooperative Oncology Group (ECOG) Performance Status (PS) ≤ 1. The primary endpoint is the confirmed objective response rate (ORR). The target sample size of the PURSUIT trial is 50 patients. Biomarker analyses will be performed in parallel using the OncoBEAM RAS CRC kit and a next-generation sequencing-based ctDNA analysis (Guardant360).

**Discussion:**

Our trial aims to confirm the clinical benefit of anti-EGFR mAb rechallenge therapy in patients with plasma *RAS* negative. Moreover, through biomarker analyses, our trial will shed light on which patients would benefit from rechallenge in addition to being plasma *RAS* negative.

**Trial registration:**

The REMARRY study: UMIN, UMIN000036424. Registered date: April 5, 2019. The PURSUIT trial: jRCT, jRCTs031190096. Registered date: October 1, 2019.

## Background

Anti-epidermal growth factor receptor (EGFR), monoclonal antibodies (mAbs), panitumumab, and cetuximab are key standard drugs for patients with metastatic colorectal cancer (mCRC) with *RAS* wild-type tumors [1–4], achieving a median overall survival (OS) of approximately 30 months [1, 2, 5, 6]. Recently, the potential efficacy of rechallenge with anti-EGFR mAbs in a later setting for patients who had benefited from previous anti-EGFR mAb therapy has been suggested in retrospective and prospective studies [7–15]. The CRICKET trial, a single-arm phase II trial of rechallenge with cetuximab in 28 patients with a response to previous anti-EGFR mAbs, demonstrated a promising objective response rate (ORR) of 21% [11], whereas the Japanese phase II JACCRO-CC-08 and -09 trials showed limited efficacy of rechallenging anti-EGFR mAbs, with an ORR of 2.9–8.3% [13].

Plasma *RAS* status in circulating tumor DNA (ctDNA) is gaining attention as a novel predictive biomarker for the efficacy of rechallenging anti-EGFR mAbs. In the CRICKET trial, an enhanced ORR of 30% and longer progression-free survival (PFS) was observed in patients without *RAS* mutations in ctDNA just before the rechallenge [11]. Moreover, in a combined analysis of the JACCRO-CC-08 and -09 trials, negative for *RAS* mutations in ctDNA was associated with improved PFS and OS in rechallenge therapy with anti-EGFR mAbs [13]. Although post hoc analyses in clinical trials have indicated that plasma *RAS* status potentially predicts the efficacy of rechallenge therapy with anti-EGFR mAbs, the utility of liquid biopsy has not been prospectively validated. Furthermore, the appropriate mutant allele frequency (MAF) cut-off level in *RAS* mutations has not been established because a different cut-off had been adopted in each post hoc analysis.

This trial is designed to prospectively monitor plasma *RAS* status in patients experiencing initial response, followed by disease progression with prior chemotherapy containing anti-EGFR mAbs, and to evaluate the efficacy of rechallenge therapy with panitumumab plus irinotecan in patients negative for *RAS* mutations in ctDNA.

## Methods/design

### Overall trial design

**Fig. 1:**
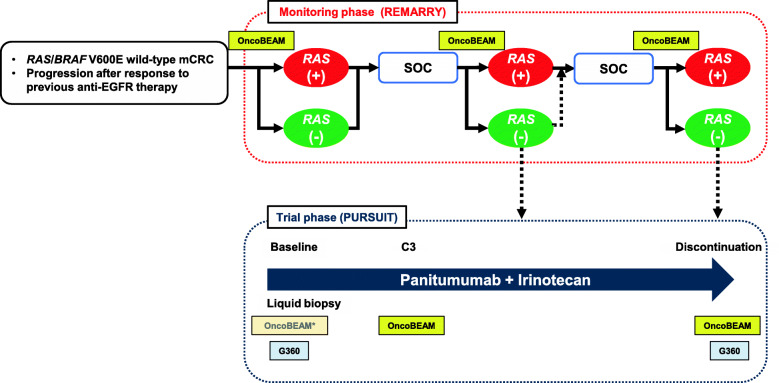
Overall trial design. Liquid biopsies for OncoBEAM RAS CRC kit and/or Guardant360 will be performed in the PURSUIT trial at baseline, cycle 3, and after discontinuation of protocol treatment. C3: Cycle 3; G360: Guardant360; OncoBEAM: OncoBEAM RAS CRC kit; SOC: Standard of care. *Substitution of the result just before enrollment

This trial is composed of 2 phases: a monitoring phase (REMARRY) and a trial phase (PURSUIT). The overall trial design is shown in Fig. 1.

### Monitoring phase (REMARRY)

The REMARRY study prospectively monitors plasma *RAS* status after refractory to anti-EGFR therapy in mCRC patients with *RAS*/*BRAF* V600E wild-type tumors in a tumor tissue sample who have progressed after a complete or partial response to previous anti-EGFR mAb therapy, which aims to evaluate the dynamics of plasma *RAS* status. Plasma *RAS* status is measured at disease progression during subsequent therapies, using a highly sensitive digital polymerase chain reaction (PCR) OncoBEAM RAS CRC kit in a central laboratory (Sysmex, Japan).

### Trial phase (PURSUIT)

The PURSUIT trial is a multicenter, single-arm phase II trial which assesses the efficacy and safety of rechallenge therapy with panitumumab plus irinotecan in patients with plasma *RAS* negative (defined as plasma MAF of all *RAS* ≤ 0.1%) in the REMARRY study.

#### Patient

Key eligibility criteria include *RAS*/*BRAF* V600E wild-type mCRC in tumor tissue refractory or intolerant to fluoropyrimidine, oxaliplatin, and irinotecan; progression after a complete or partial response to previous anti-EGFR mAb therapy; plasma *RAS* negative (MAF of all *RAS* ≤ 0.1%) within 28 days prior to enrollment; 4 months or more between the last administration of previous anti-EGFR mAbs and the start of protocol treatment; and Eastern Cooperative Oncology Group (ECOG) Performance Status (PS) ≤1. Details of the eligibility criteria are listed in Table 1.

**Table 1:** Eligibility criteria for the PURSUIT trial

Inclusion criteria	Exclusion criteria
1. Unresectable colorectal cancer pathologically diagnosed as adenocarcinoma 2. *RAS* (*KRAS*/*NRAS*) and *BRAF* V600E wild-type in tumor tissue sample 3. Patients intolerant or refractory to chemotherapy, including fluoropyrimidine, oxaliplatin, and irinotecan 4. Complete or partial response to previous chemotherapy, including anti-EGFR mAb (cetuximab or panitumumab) according to RECIST version 1.1 5. Documentation of progression to previous anti-EGFR therapy within 2 months after last anti-EGFR mAb administration 6. Patients negative for *RAS* mutations in ctDNA using OncoBEAM RAS CRC kit within 28 days before enrollment in the REMARRY study 7. Four months or more between the last administration of previous anti-EGFR mAbs and the start of protocol treatment 8. Measurable disease according to RECIST version 1.1 9. ECOG PS 0 or 1 10. Age 20 years or older 11. Adequate major organ function assessed within 14 days before enrollment: a. Neutrophil count ≥1500/mm3 b. Platelet count ≥75,000/mm3 c. Hemoglobin ≥9.0 g/dL d. ALT and AST ≤100 IU/L (≤ 200 IU/L for patients with liver metastasis) e. Serum creatinine ≤1.5 mg/dL 12. Life expectancy of at least 12 weeks 13. Written informed consent obtained	1. Severe comorbidity. a. Synchronous active malignancies b. Uncontrolled brain metastasis or leptomeningeal metastasis c. Active infectious disease d. Uncontrolled ascites, pleural effusion, or pericardial effusion requiring continued drainage e. Uncontrolled diabetes mellitus or hypertension f. Myocardial infarction, severe/unstable angina pectoris, symptomatic congestive heart failure of New York Heart Association Class III or IV within 6 months before the enrollment g. Psychiatric diseases or psychiatric symptoms considered as difficult to enroll in a clinical trial 2. Underwent one of following treatments before protocol treatment: a. Extensive surgery within 4 weeks b. Colostomy/ileostomy within 2 weeks c. Chemotherapy within 2 weeks d. Radiation therapy within 2 weeks 3. CTCAE Grade ≥ 2 adverse events due to previous therapy, which are not recovered 4. History of severe infusion reactions to anti-EGFR mAbs 5. Intolerant to previous irinotecan therapy 6. Comorbidity or history of severe pulmonary disease 7. Men/women who are unwilling to avoid pregnancy; women who are pregnant or breastfeeding; women with a positive pregnancy test 8. Known active HCV or HIV infection 9. Any other patients who are regarded as inadequate for trial enrollment by investigators

*ALT* alanine aminotransferase, *AST* aspartate transaminase, *CTCAE* Common Terminology Criteria for Adverse Events, *ctDNA* circulating tumor DNA, *ECOG* Eastern Cooperative Oncology Group, *EGFR* epidermal growth factor receptor, *HCV* hepatitis C virus, *mAb* monoclonal antibody, *PS* Performance Status, *RECIST* Response Evaluation Criteria in Solid Tumors

#### Treatment

Patients will receive panitumumab 6 mg/kg plus irinotecan 150 mg/m^2^ biweekly until progressive disease, unacceptable toxicity, informed consent withdrawal, or patient’s death. The starting dose of irinotecan can be reduced to 120 mg/m^2^ or 100 mg/m^2^ according to adverse events during previous irinotecan therapy.

#### Outcomes and statistical considerations

The primary endpoint of the PURSUIT trial is the confirmed ORR, defined as the proportion of patients who achieve confirmation of complete or partial response by the investigator’s assessment with a minimum interval of 4 weeks. The secondary endpoints include PFS, time to treatment failure, duration of response, OS, disease control rate, and incidences of adverse events. Efficacy will be evaluated according to Response Evaluation Criteria in Solid Tumors (RECIST) version 1.1, using computed tomography at 6 and 12 weeks after the start of treatment and every 8 weeks thereafter. The ORR threshold is set at 10%, based on the results of previous clinical rechallenge trials with anti-EGFR mAbs [11, 13–15]. The required sample size was calculated as 45, with an ORR of 25% deemed promising (one-sided α, 0.05; β, 0.15) [11]. Considering drop-outs and ineligible patients, the target sample size is 50 patients. The primary endpoint will be analyzed in a full analysis set (PURSUIT-FAS) of all patients enrolled in the PURSUIT trial, receiving at least one dose of protocol treatment and satisfying all the inclusion and exclusion criteria. All statistical analyses will be performed using SAS software, version 9.2 (SAS Institute).

#### Biomarker analysis

Liquid biopsies will be performed in the PURSUIT trial at baseline, cycle 3, and after discontinuation of protocol treatment. The ctDNA will be analyzed using a highly sensitive digital PCR method, OncoBEAM RAS CRC kit, and a targeted next-generation sequencing, Guardant360. Figure 1 shows at which point each analysis is performed. OncoBEAM RAS CRC kit, which uses beads, emulsion, amplification, magnetics (BEAMing) digital PCR technology, detects 34 mutations in *KRAS*/*NRAS* codons 12, 13, 59, 61, 117, and 146 in plasma [16]. This test is an in vitro diagnostic test, CE-marked in Europe and approved by the Pharmaceuticals and Medical Devices Agency in Japan to detect *RAS* mutations in ctDNA derived from mCRC. Several prospective and retrospective studies comparing *RAS* status as determined by BEAMing in plasma and the tissue reference method have reported high concordance rates, from 86.4 to 93.3% [17–20]. Guardant360 is a hybrid capture-based next-generation sequencing panel of ctDNA by Guardant Health, which is a Clinical Laboratory Improvement Amendments-certified, College of American Pathologists-accredited, New York State Department of Health-approved laboratory, as previously described [21]. Briefly, Guardant360 detects 74 gene alterations, including single nucleotide variants, indels, amplifications, and fusions, with a reportable range of ≥0.04, ≥0.02, ≥0.04%, and ≥ 2.12 copies, respectively.

### Integrated analysis

Data on baseline characteristics and clinical outcomes will be collected on patients enrolled in the REMARRY study receiving rechallenge with anti-EGFR mAb in clinical practice from the PURSUIT trial (clinical practice set [plasma MAF of all *RAS* > 0.1%]). An integrated analysis, including PURSUIT-FAS (MAF ≤0.1%) and the clinical practice set (MAF > 0.1%), will be performed to determine a clinically significant plasma *RAS* MAF cut-off value.

### Trial organization

This trial is supported by a nationwide cancer biomarker screening project, SCRUM-Japan [22]. Participating institutions include 28 core centers in Japan.

## Discussion

Post hoc analyses of clinical trials have indicated the clinical significance of plasma *RAS* status at baseline as a predictive biomarker for the efficacy of rechallenge with anti-EGFR mAbs in patients with mCRC. Beyond these data, our trial will reveal some important points to select patients who benefit from rechallenge with anti-EGFR mAbs.

First, our trial’s findings will enable us to estimate the optimal cut-off value for *RAS* MAF in ctDNA associated with the efficacy of rechallenge with anti-EGFR mAbs. Given the cut-off values have varied in previous reports, the optimal value remains unclear. Although the absolute cut-off value is defined as 0.1% in the PURSUIT trial based on the previous retrospective or *post-hoc* analyses [14, 23], integrated analysis of rechallenge with anti-EGFR mAbs in PURSUIT-FAS (MAF ≤0.1%) and the clinical practice set (MAF > 0.1%) will be performed to determine the optimal cut-off value of plasma *RAS*.

Second, our trial could shed more light on the relationship of temporal-spatial tumor heterogeneity and rechallenge efficacy. Previous reports have focused mainly on plasma *RAS* status just before rechallenge; the role of plasma *RAS* status just after refractory to previous anti-EGFR therapy as a biomarker for rechallenge remains unknown. Moreover, it is unclear whether acquired alterations other than *RAS* mutations, including *BRAF*, *EGFR*, *HER2, MET*, and *PIK3CA,* affect the efficacy of rechallenge with anti-EGFR mAbs [24–28]. Our trial monitors serial ctDNA status from just after refractory to the previous anti-EGFR therapy using OncoBEAM RAS CRC kit and a plasma-targeted next-generation sequencing panel (Guardant360), allowing us to reveal how the dynamics of *RAS* mutations and other acquired alterations influence rechallenge efficacy.

Third, our trial could also clarify the significance of clinical factors in a plasma *RAS*-negative population. Although clinical factors, including the anti-EGFR mAb-free interval and PFS for previous anti-EGFR therapy, have been assessed in patients without a plasma *RAS* test, it is unknown whether clinical factors still predict the efficacy of rechallenge with anti-EGFR mAbs in patients with plasma *RAS* negative. Our trial will help patient selection by using clinical factors and molecular markers to enhance the efficacy of rechallenge with anti-EGFR mAbs in patients with mCRC.

## Data Availability

Not applicable.

## Abbreviations

ctDNA: Circulating tumor DNA
ECOG: Eastern Cooperative Oncology Group
EGFR: Epidermal growth factor receptor
mAb: Monoclonal antibody
MAF: Mutant allele frequency
mCRC: Metastatic colorectal cancer
ORR: Objective response rate
OS: Overall survival
PFS: Progression-free survival
